# Using Zoos as Sentinels for Re-Emerging Arboviruses: Vector Surveillance during an Outbreak of Epizootic Hemorrhagic Disease at the Minnesota Zoo

**DOI:** 10.3390/pathogens12010140

**Published:** 2023-01-14

**Authors:** Bethany L. McGregor, Lindsey M. Reister-Hendricks, Cale Nordmeyer, Seth Stapleton, Travis M. Davis, Barbara S. Drolet

**Affiliations:** 1 Arthropod-Borne Animal Diseases Research Unit, Center for Grain and Animal Health Research, USDA-Agricultural Research Service, Manhattan, KS 66502, USA; 2 Conservation Department, Minnesota Zoo, Apple Valley, MN 55124, USA; 3 Department of Fisheries, Wildlife and Conservation Biology, University of Minnesota, St. Paul, MN 55108, USA

**Keywords:** *Culicoides*, epizootic hemorrhagic disease virus, orbivirus, surveillance, vector-borne disease

## Abstract

Vector-borne disease prevalence is increasing at a time when surveillance capacity in the United States is decreasing. One way to address this surveillance deficiency is to utilize established infrastructure, such as zoological parks, to investigate animal disease outbreaks and improve our epidemiological understanding of vector-borne pathogens. During fall 2020, an outbreak of epizootic hemorrhagic disease (EHD) at the Minnesota Zoo resulted in morbidity and seroconversion of several collection animals. In response to this outbreak, insect surveillance was conducted, and the collected insects were tested for the presence of epizootic hemorrhagic disease virus (EHDV) by RT-qPCR to better understand the local transmitting vector populations responsible for the outbreak. Six pools of *Culicoides* biting midges were positive for EHDV, including three pools of *Culicoides sonorensis*, two pools of *Culicoides variipennis*, and a pool of degraded *C. variipennis* complex midges. All three endemic serotypes of EHDV (1, 2, and 6) were detected in both animals and midge pools from the premises. Despite this outbreak, no EHDV cases had been reported in wild animals near the zoo. This highlights the importance and utility of using animal holding facilities, such as zoos, as sentinels to better understand the spatio-temporal dynamics of pathogen transmission.

## 1. Introduction

The prevalence of vector-borne diseases is increasing in the United States and around the world, necessitating increased surveillance to prevent outbreaks and potentially save human and animal lives [1,2]. Despite this, vector-borne disease surveillance capacity in the US is decreasing, and surveillance is often seen as an easy area for funding reduction and cutbacks [3,4]. This combination of decreased surveillance capacity and increasing disease prevalence makes it harder to detect and mitigate outbreaks before they lead to morbidity and mortality. Therefore, finding ways to use the existing infrastructure and personnel already monitoring vector populations near susceptible animals, such as zoological parks or conservation areas [5], provides an opportunity to increase surveillance more broadly and inform spatio-temporal pathogen dynamics beyond our current methods.

Epizootic hemorrhagic disease virus (EHDV) is an orbivirus in the Reoviridae family that causes a hemorrhagic disease in white-tailed deer (*Odocoileus virginianus*), mule deer (*Odocoileus hemionus*), and pronghorn antelope (*Antilocapra americana*). The clinical signs can be dramatic in these species, including fever, respiratory distress, edema of the head and neck, and internal hemorrhaging, as well as possible chronic presentations such as emaciation and hoof sloughing [6]. Other species, including cattle, tend to have mild or subclinical infections affecting production, such as decreased milk production in dairy cows [7], and can potentially serve as animal reservoirs for the virus. Furthermore, other species can maintain an EHDV viremia while displaying no observable pathology, such as elk [8], that may contribute to outbreaks as silent reservoirs. It is largely unclear what role exotic hoofstock may play in the epizootiology of EHDV in North America, although there is evidence that species such as fallow deer (*Dama dama*), Sika deer (*Cervus nippon*), Père David’s deer (*Elaphurus davidianus*), Dama gazelle (*Nanger dama*), and blackbuck antelope (*Antilope cervicapra*) do seroconvert [9,10].

The only fully confirmed vector of EHDV in North America is *Culicoides sonorensis* [11]. This species is abundant throughout the western USA, with sporadic populations also present in the eastern and southern USA [12,13], and is associated with extremely organically enriched larval habitats, especially dairy wastewater ponds [14]. Although *C. sonorensis* is considered the major vector in North America, other *Culicoides* species have been identified as suspected vectors, such as *C. debilipalpis, C. stellifer*, and *C. venustus* [15,16]. This is due to direct evidence, including virus detection in field-collected insects, and/or anecdotal evidence, such as a great abundance of suspected vectors on the outbreak landscape in the absence of *C. sonorensis*, especially in areas where EHDV-susceptible hosts are present [16,17]. The vector community responsible for the transmission of EHDV in the northern and eastern USA and in areas where *C. sonorensis* is less abundant is currently unresolved.

EHDV has historically been found throughout the western, southeastern, and central USA, although the virus has experienced a northern spread into novel areas of the northeastern and upper midwestern USA within the past 20 years [18,19]. Outbreaks of the virus tend to occur in the late summer into fall and can follow several epidemiological patterns depending on the geographic location and endemicity of the pathogen. In the southeastern USA, where the virus is endemic and circulates almost annually, outbreaks are typically mild. However, in areas where outbreaks are less frequent or newly occurring, epidemics can lead to high morbidity and mortality [19]. EHDV is a relatively new pathogen in Minnesota, with the first occurrence of the disease in white-tailed deer detected in a herd of captive individuals in Goodhue County in 2018. A year later, EHDV was confirmed in four wild white-tailed deer in Stearns County and in additional deer in Houston County (Minnesota Department of Natural Resources, unpublished data).

In September 2020, the Minnesota Zoo, situated on a roughly 500-acre campus in east-central Minnesota, experienced an outbreak of EHD among collection animals. The virus was first detected in an outdoor herd of caribou (*Rangifer tarandus*; serotypes 1, 2, and 6) after several individuals began showing clinical signs consistent with the disease and ultimately resulted in the acute mortality of four individuals. Additional collection animals were subsequently tested and confirmed as positive, including five of six bison (*Bison bison*), two of five pronghorn antelope (*Antilocapra americana*), two of three moose (*Alces alces*), and one of one black-tailed gazelle (*Gazella subgutturosa*). This outbreak provided a unique opportunity to improve our understanding of vector-borne transmission to native and nonnative animal species. Specifically, to gain insights into potential vector species transmitting EHDV in the area, we trapped and sorted *Culicoides* midges and tested pools for the virus by RT-qPCR. 

## 2. Materials and Methods

### 2.1. Insect Collections and Pooling

**Figure 1:**
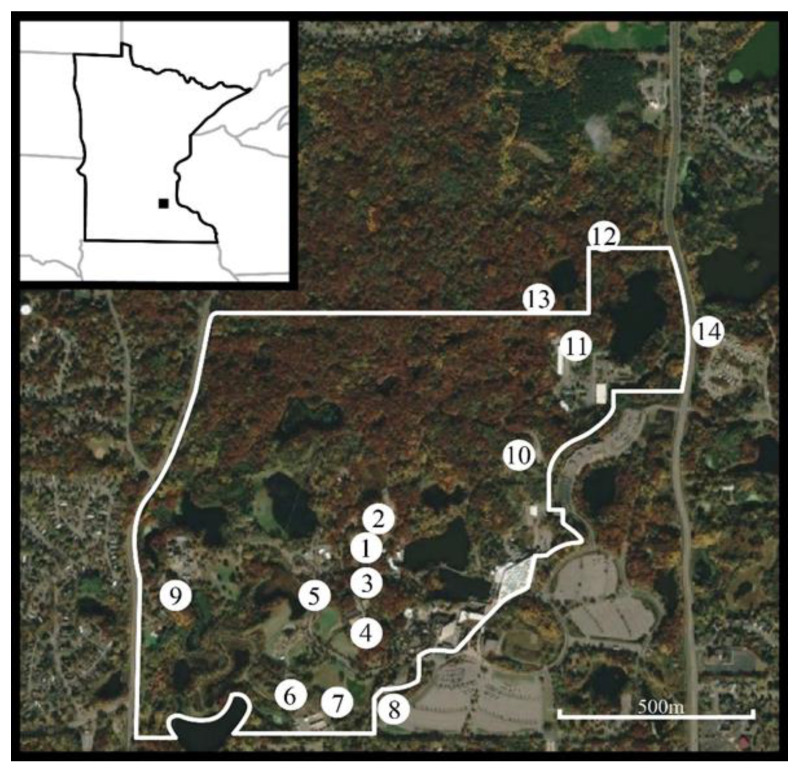
Fall 2020 biting insect trapping locations on the Minnesota Zoo campus as indicated by numbered circles. The white line indicates the zoo’s exterior-most perimeter fence which serves as a barrier to white-tailed deer. (1) Caribou Exhibit; (2) Caribou Holding; (3) Moose Exhibit; (4) Drainage area between the Takin and the Pronghorn Exhibits; (5) Bactrian camel Exhibit; (6) Asian wild horse holding; (7) Bison holding; (8) Exterior location; (9) West compost pad; (10) East compost pad; (11) Quarantine holding area; (12) Lebanon Hills trap 1; (13) Lebanon Hills trap 2; (14) Lebanon Hills trap 3. Locations 8, 12, 13, and 14 are outside of the zoo perimeter fence. Inset: Minnesota Zoo’s location in Apple Valley, MN. Aerial image: 2022 CNES/Airbus: collected 12 October 2020. Accessed via Google Earth Pro, December 2022.

In response to the EHD outbreak at the Minnesota Zoo, the zoo staff began sampling for biting insects to document the occurrence and spatio-temporal distribution of potential vector species. We conducted biting insect surveillance using CDC light traps (Clarke, St Charles, IL, USA) fitted with a 150 mA incandescent bulb, baited with 1.5 kg of dry ice and suspended at 1.5 m. Trapping took place during eight evenings between 17 September 2020 and 3 November 2020 starting about two weeks after the first caribou began exhibiting clinical signs. Ten trapping locations were selected based on their proximity to the caribou and other ungulate taxa in the collection that could potentially contract EHDV (Figure 1). We deployed an additional four trap locations within five meters outside the zoo’s perimeter fence (where wild white-tailed deer are not excluded). All trap locations were selected for having a consistently wet and organically rich soil. We deployed traps between 17:00 and 18:00, after the zoo was closed to the public, and collected their contents the following day between 07:00 and 08:30. Immediately after collecting the trap contents, we pooled the *Culicoides* specimens in groups of ten by trapping location, placed them into ethanol, and stored them in a −80 °C freezer.

A subset of the collected insects was randomly selected for EHDV screening, ensuring representation from different sampling dates and locations, and sent to the Arthropod-Borne Animal Diseases Research Unit at the Center for Grain and Animal Health Research in Manhattan, Kansas, where they were identified to species using morphological characteristics [13,20]. The specimens were sorted according to species, collection date, and collection location into pools of 11 or fewer individuals. All midges were screened for observable blood in the gut to prevent the detection of the virus in host blood rather than midge tissues. No blood-engorged midges were identified, so no specimens needed to be excluded. The midges were rinsed twice with ultrapure water to remove ethanol prior to placement into 500 µL of antibiotic medium in 1.5 mL microcentrifuge tubes. The pooled midges were stored at −80°C until further processing.

### 2.2. Total RNA Extraction

Frozen, sorted insect pools were thawed on wet ice and homogenized on a Bead Ruptor Elite (Omni International, Kennesaw, GA, USA) for 2 min at 3.1 m/s, repeated twice. The samples were then returned to wet ice during processing. 

Next, 1 mL of TRIzol (Invitrogen, Thermo Fisher, Waltham, MA, USA) was added to each tube. The tubes were then vortexed briefly to mix and allowed to rest at room temperature for 5 min before they were centrifuged for 5 min, 4 °C, at 12,000× *g*. The supernatant was transferred into a new tube, and 200 µL of trimethylene bromochloride (BCP) (Sigma Aldrich, St. Louis, MO, USA) was added for phase separation. The tubes were inverted by hand for 20 s to mix their contents, then incubated for 5 min at room temperature. The tubes were then centrifuged at 12,000× *g* for 15 min at 4 °C, the aqueous phase was transferred into a new tube, and 500 µL of 100% isopropanol was added. The tubes were again inverted by hand to mix and incubated for 10 min at room temperature. 

The samples were centrifuged at 12,000× *g* for 10 min at 4 °C, the supernatant was discarded, and the remaining pellet was washed with 1 mL of 75% ethanol and vortexed briefly. The tubes were centrifuged at 7500× *g* for 5 min at 4 °C, and the pellet was washed a second time as above. After the final wash with ethanol, the tubes were allowed to air dry before the pellets were dissolved in 50 µL of DEPC water (Invitrogen, Thermo Fisher, Waltham, MA, USA) and incubated in a heat block at 56 °C for 10 min. The samples were stored at −80 °C.

### 2.3. cDNA Synthesis

Following the methods described previously [21], 200 ng of total RNA was used as template in a mastermix of qScript XLT cDNA SuperMix (Quantabio, Beverly, MA, USA), including a mix of random hexamers and oligo(dT) primers, for a total reaction volume of 20 µL as per the manufacturer’s amplification instructions. 

### 2.4. EHDV RNA Detection by RT-qPCR 

The reactions were performed in technical triplicates per primer set using the iQ SYBR Green Supermix (Bio-Rad, Hercules, CA, USA), 5 µL of cDNA, and primers for each extraction. Primers for the conserved NS3 gene of EHDV-2 which targets all three domestic serotypes of EHDV (1, 2, and 6) were used [21], as well as midge EF1b gene primers as an internal control (Supplementary Table S1) [22,23]. For the amplification, the following temperature profile was used: 1 cycle at 95 °C for 3 min, then 40 cycles at 95 °C for 10 s and 60 °C for 45 s. This was followed by a standard melt curve from 55 to 95 °C, 2–5 s per step on a Bio-Rad CFX real-time PCR machine (Bio-Rad, Hercules, CA, USA). The samples were analyzed with Bio-Rad CFX Maestro qPCR software and were determined positive only if the EF1b wells resulted in a threshold cycle (C_t_) between 21 and 25 and the NS3 samples were positive in at least two of the three technical replicates and produced a smooth, single-peak melt curve with a fluorescent maximum at 80 °C [21]. All reaction plates included a no-template control containing molecular-biology-grade water.

### 2.5. EHDV Serotyping RT-qPCR

The samples that were positive during the initial EHDV detection were then tested for serotypes 1, 2, and 6. The reactions were performed in technical triplicates using the Superscript III/ Platinum Taq One-Step qRT-PCR Kit (Invitrogen, Waltham, MA, USA). The primers and probes (LGC Biosearch Technologies, Petaluma, CA, USA) were specific to the VP2, seg-2 region of each virus serotype [24] (Supplementary Table S1). The samples were amplified on a Bio-Rad CFX OPUS real-time PCR machine under the following conditions: 55 °C for 30 min, 95 °C for 10 min, and 50 cycles at 95 °C for 30 s and 60 °C for 1 min. The data were analyzed using Bio-Rad CFX Maestro qPCR software.

### 2.6. Field Infection Rate Calculations

The minimum infection rate (MIR) was calculated for all species with positive pools. The MIR was calculated by taking the number of positive pools, dividing by the total number of midges tested, and multiplying by 1000 to represent the number of positive midges per 1000 in the population. The MIR is considered a conservative estimate of infection rate since it assumes that each positive pool is caused by a single positive individual in the pool.

## 3. Results

We detected *Culicoides* biting midges at 5 of the 14 trapping locations on and near the Minnesota Zoo grounds during eight sampling nights (Table 1). We documented the highest relative abundances in the caribou exhibit, their holding area, and in the moose exhibit (~90 m from the caribou exhibit trap location). The total sample of *Culicoides* collected at the caribou exhibit during the survey window was 10 times greater than that obtained combining the samples from all other sites. 

A total of 964 *Culicoides* were placed into 103 pools of 11 or fewer individuals, including 1 pool of *Culicoides crepuscularis* (n = 1 individual), 16 pools of *Culicoides sonorensis* (n = 150 individuals), 2 pools of *Culicoides stellifer* (n = 16 individuals), 75 pools of *Culicoides variipennis* (n = 715 individuals), and 9 pools of *C. variipennis* complex individuals (n = 82 individuals) (Table 2). The pools of *C. variipennis* complex midges were identifiable to the species complex but were too degraded for further identification to the species level.

Of 103 total pools tested, six pools tested positive for EHDV (Table 2). These included 3 of 16 pools of *Culicoides sonorensis*, indicating an MIR of 20 infected individuals per 1000 in the population, and 2 of 75 pools of *C. variipennis*, with an MIR of 2.8 infected individuals per 1000. The final positive pool was one pool of the nine containing degraded *C. variipennis* complex individuals. Because a species-level identification was not possible for these individuals, no MIR was calculated for this positive pool.

All six positive pools were collected from the caribou exhibit at the Minnesota Zoo, but not on the same date. Three positive pools (*C. sonorensis, C. variipennis*, degraded *C. variipennis* complex) were collected on 25 September, two pools (*C. sonorensis, C. variipennis*) were collected on 26 September, and the remaining positive pool from *C. sonorensis* was collected on 1 October. 

The serotype detection of the positive pools revealed that all three endemic EHDV serotypes were detected in the collected insects (Table 3). Pools containing single and multiple serotypes were found for both species, and EHDV-6 was the most common serotype detected, found in pools of both *C. sonorensis* and *C. variipennis* as well as the degraded *C. variipennis* complex pool. There were too few positives to determine statistically whether any of the serotypes were associated with a certain species.

## 4. Discussion

Outbreaks of EHDV can cause significant morbidity and mortality to native and exotic wildlife and livestock in the USA. Understanding the vector and host dynamics of EHDV outbreaks can help us better prepare for future outbreaks and mitigate their impacts. In response to an outbreak of EHD at the Minnesota Zoo in 2020, insects were collected and tested for EHDV to improve our understanding of transmission dynamics and epizootiology. We detected six EHDV-positive pools from *Culicoides* midges collected during the outbreak, including pools of *C. sonorensis, C. variipennis*, and *C. variipennis* complex individuals, and all three endemic EHDV serotypes were detected. All positive pools were collected at a single location, the caribou exhibit, which incidentally had the highest relative abundance of *Culicoides* midges.

*Culicoides sonorensis* is the only fully confirmed EHDV vector in North America, so the positive pools detected from this species are unsurprising. However, the positive pools of *C. variipennis* are novel. This species, which is closely related to *C. sonorensis*, has previously been considered an unlikely EHDV vector. One complicating factor, however, is the ever-evolving taxonomical treatment of this complex. Historically, *C. variipennis* was treated as a species with several subspecies (including *C. variipennis sonorensis*) [25]. A taxonomic reassessment in 2000 raised *C. sonorensis* to the species level based on population sympatry and electrophoretic data [13]. For this reason, much of the available early literature on vector competence is presented using the species name *C. variipennis*, and the true identity of the specimens is unclear [26]. Despite this lack of clarity, it seems likely that *C. variipennis* retains some degree of competence for virus transmission in common with *C. sonorensis*, based on the present data. In addition, vesicular stomatitis virus Indiana was detected from a *C. variipennis* pool in Kansas [27], suggesting that this species may be a putative vector of several midge-borne viruses.

While it is notable that *C. variipennis* pools were EHDV-positive, it is also important to note that the MIR was 10× lower for this species than for *C. sonorensis*. The high MIR for *C. sonorensis* suggests that this species drove the outbreak as a primary vector, while *C. variipennis* contributed to the outbreak to a lesser or secondary extent. However, in calculating metrics such as vectorial capacity, abundance is an important variable [28]. In the present study, the abundance of *C. variipennis* on the landscape was higher than that of *C. sonorensis*, a dynamic that may balance out a lower susceptibility to infection in *C. variipennis*. The volume of midges, primarily *C. variipennis*, detected at the caribou exhibit relative to other sampling locations could reflect this dynamic. Additional studies on the vector competence of *C. variipennis* are needed. Furthermore, while other vertebrate species at the zoo were positive for EHDV, the caribou reported the only mortalities and seemed to bear the brunt of the midge abundance collected. A 1991 report by the Alaska Department of Fish and Game found that while some EHDV seroconversion was documented in caribou, clinical cases were not identified, and this virus seemed rare in caribou populations [29]. The range of *C. sonorensis* is not thought to extend into the native range of caribou. However, in situations where these vertebrates are brought into closer contact with competent vectors, it appears they may be more susceptible to infection than previously thought. Additional studies investigating the susceptibility of caribou to EHDV and their potential role in the epizootiology of this pathogen, especially in light of climate change and shifting ranges of both vertebrates and invertebrate populations, are suggested.

*Culicoides sonorensis* and *C. variipennis* were by far the most abundant midge species collected, but another species present in the collections, *C. stellifer*, has also been implicated as a potential EHDV vector. EHDV-positive pools of *C. stellifer* have been collected in Florida [15], and several studies throughout the southeastern USA have noted a high abundance of this species during EHD outbreaks [16,17,30]. The low abundance of this species collected in this study may reflect either lower abundance in the northern reaches of the species range or less availability of preferred habitats on the zoo grounds. While *C. sonorensis* and *C. variipennis* have historically been more associated with livestock and managed habitats, *C. stellifer* is often collected in sylvatic habitats [26]. Furthermore, it is unclear whether the midge community collected during this study is typical for this area or whether any recent changes in the midge community could have facilitated the EHD outbreak. This further reinforces the need for long-term sentinel animal sites to provide not only surveillance for virus outbreaks, but also contextual information on local vector communities. Finally, different populations within the same species can also have different competence for viruses, which has been seen in mosquito-borne virus systems [31,32]. It is possible that *C. stellifer* is a competent vector in some parts of its range but not in others. 

Previous studies have shown that the presence of high densities of diverse vertebrate hosts associated with locations such as big game preserves and zoos can lead to high abundance and diversity of adult *Culicoides* and other biting Diptera [33,34,35]. This is likely due to the increase in attractive volatiles from hosts and the availability of diverse, numerous oviposition sites and larval habitats. *Culicoides* larvae are considered semi-aquatic and develop in moist substrates, often along the soil–water interface of water features such as ponds and troughs that are common within animal holding facilities. While *Culicoides* are capable of flight up to a 2–4 km total distance [36,37], most individuals do not disperse more than 1 km [36], especially when the immediate area provides food sources and breeding habitats. Due to the high abundance of midges often collected at zoos and other animal holding preserves [33,34,35] and the propensity of *Culicoides* to disperse to relatively short distances overall, these types of establishments can act as unique sentry points to detect outbreaks that may not be as readily detected in the surrounding wild populations. No cases of EHDV were reported in the wild ungulate populations surrounding the Minnesota Zoo in 2020, although there was a documented outbreak elsewhere in Minnesota in 2019. Without these Minnesota Zoo collections, our epidemiological understanding of EHDV transmission dynamics in this ecosystem may be flawed by the assumption that the virus was not present in situ in 2020. Vector surveillance is often conducted by state agencies or academic groups in response to reports of sick or dead animals. This provides information on potential vector species and on the breadth of an outbreak but is reactive in nature. Furthermore, some academic groups conduct ongoing vector surveillance projects, but these are often limited in scope, both spatially and temporally. Sentinel chicken sites are used in some areas of the USA for the detection of mosquito-borne pathogens that can infect birds, such as West Nile virus and St. Louis encephalitis virus [38]. These sentinel sites provide an important early warning system to inform the potential for outbreaks in human populations and to guide mosquito control efforts. However, the maintenance of designated sentinel houses for mammalian hosts is challenging, especially for the detection of pathogens that infect large ungulates. Therefore, establishing a network of sentinel animal holding facilities would allow the quantification of the spatio-temporal movement of mammal pathogens in a way that is not possible using current methods.

One important caveat for this study is the recent elevation of *Culicoides albertensis* to the species level. This species had previously been synonymized with *C. sonorensis* but was re-elevated to the species status after the collection of compelling genetic data [39]. Due to the close morphological similarity between *C. albertensis* and other members of the *C. variipennis* complex, it is possible that this species was present within the study area but was unable to be morphologically distinguished. The processing of pools for viral RNA extraction prevented diagnostic molecular determinations of the species. An additional limitation of this study is the use of just RT-qPCR for the detection of virus-positive pools without confirmation of viable virus through cell culture. The midges were shipped in ethanol, which may have impacted the viability of any virus present. While we did detect EHDV nucleic acids in the pools, it is unclear whether the detected virus was viable without this confirmatory step. Finally, even if viable virus was able to bypass the midgut escape barrier to infect body tissues, we cannot determine with this evidence whether infection of the salivary glands, and therefore transmission potential, occurred.

In this study, we identified EHDV-positive pools of two species of *Culicoides* using a zoo as a sentinel site. These results contribute to our knowledge of the EHDV vector species and transmission dynamics in Minnesota, a state for which little information is available since the first EHDV detection occurred in 2018. Understanding which species may contribute to and drive disease outbreaks can help us to better monitor and mitigate disease threats. Furthermore, these EHDV-positive pools fill in a gap in the spatio-temporal landscape, since no positives were detected in surrounding wild ungulate populations in 2020, underscoring the utility of using animal holding facilities such as zoos as sentinel sites for arboviral detections. 

## Figures and Tables

**Table 1:** Trapping results associated with vector surveillance conducted at 14 sites at and near the Minnesota Zoo from 18 September 2020 to 4 November 2020. Numbers indicate the total number of *Culicoides* biting midges collected during that sampling session. Dates reflect the mornings that traps were collected.

Trap Location	18 September 2020	19 September 2020	25 September 2020	26 September 2020	1 October 2020	8 October 2020	15 October 2020	4 November 2020	Total
(1) Caribou Exhibit	11	191	251	1226	72	68	3	0	1822
(2) Caribou Holding	-	-	77	49	1	4	0	0	131
(3) Moose Exhibit	0	0	1	38	0	0	0	0	39
(4) Takin/Pronghorn Exhibit	0	0	0	0	0	0	0	0	0
(5) Bactrian Camel Exhibit	-	0	1	1	0	0	0	-	2
(6) Asian Wild Horse Holding	0	0	0	-	0	0	-	-	0
(7) Bison Holding	-	0	1	0	1	1	-	0	3
(8) Exterior Location *	0	0	-	0	0	0	0	0	0
(9) West Compost Pad	-	0	0	-	0	0	0	-	0
(10) East Compost Pad	0	-	0	0	0	0	0	0	0
(11) Quarantine Holding Area	0	0	0	0	0	0	0	0	0
(12) Lebanon Hills Trap 1 *	0	0	0	0	0	0	0	0	0
(13) Lebanon Hills Trap 2 *	0	0	0	0	0	0	0	0	0
(14) Lebanon Hills Trap 3 *	0	0	0	0	0	-	-	0	0

* Denotes locations outside the zoo’s perimeter fence where wild white-tailed deer would have access. - Denotes nights during which sampling could not take place due to an inability to access that site or results were excluded due to a trap malfunction.

**Table 2:** *Culicoides* biting midges collected at the Minnesota Zoo during the 2020 EHDV outbreak that were randomly selected for EHDV testing. Species, total per species (N), number of pools (n ≤ 11) tested, number of positive pools detected, and calculated minimum infection rate (MIR) per 1000 individuals in the population are shown.

Species	N	# Pools	Positive Pools	MIR
*C. crepuscularis*	1	1	0	0
*C. sonorensis*	150	16	3	20
*C. stellifer*	16	2	0	0
*C. variipennis*	715	75	2	2.8
*C. variipennis* complex	82	9	1	NC *

* MIR for the *C. variipennis* complex was not calculated (NC) since species-level identifications were not made.

**Table 3:** Serotypes of EHDV positive pools collected at the Minnesota Zoo.

Positive #	Species	Pool Size	EHDV-1	EHDV-2	EHDV-6
1	*C. sonorensis*	10	N	N	Y
2	*C. sonorensis*	10	Y	N	Y
3	*C. sonorensis*	10	Y	N	N
4	*C. variipennis*	10	Y	N	Y
5	*C. variipennis*	10	N	Y	N
6	Degraded *C. variipennis* Complex	10	N	N	Y

## Data Availability

Data will be made available on Ag Data Commons and are also available from the authors by request.

## Supplementary Materials

The following supporting information can be downloaded at: https://www.mdpi.com/article/10.3390/pathogens12010140/s1, Supplementary Table S1: Primer sequences used to detect EHDV-positive samples (EHDV-2 NS3), act as a positive control (*C. sonorensis* EF1b), and determine the serotypes of the positive pools (EHDV-1, EHDV-2, and EHDV-6 VP2).
