# Supplementary material for: Using Zoos as Sentinels for Re-Emerging Arboviruses: Vector Surveillance during an Outbreak of Epizootic Hemorrhagic Disease at the Minnesota Zoo
Source: Pathogens. 2023 Jan 14;12(1):140. doi: 10.3390/pathogens12010140 (PMC9864106; doi:10.3390/pathogens12010140)
Supplement: Supplementary file 1 [file pathogens-12-00140-s001.zip › pathogens-2139929-supplementary.pdf]

Supplementary Table S1. Primer sequences used to detect EHDV-positive samples (EHDV-2 NS3), act as a positive control (*C. sonorensis* EF1b), and determine the serotypes of the positive pools (EHDV-1, EHDV-2, and EHDV-6 VP2).

Gene	Forward Primer	Reverse Primer	Probe	Citation
EHDV-2 NS3	5′-CTACCACAGCCGCAATTA-3′	5′-GCATGTAAACGAGCAAGTATT-3′	N/A	[21]
C. sonorensis EF1b	5′-ATCCGTGAAGAACGTCTCAAA-3′	5′-CATGGCTTAACTTCGAGGATG-3′	N/A	[22]
EHDV-1 VP2	5′-GAATAATTCGYTAYGAGAATAAARCYAAAG-3′	5′-TCTATGYGYCTCRTCCATTCTYGG-3′	5′-6-HEX-CAGCTGCGGTCATCTATTAGGCATC-BHQ1-3′	[24]
EHDV-2 VP2	5’ – TATGTTAAATGTATTGAATTATACG – 3’	5’ – TCTCATCCCGACCAACACT-3’	5’ – 6-HEX-CTCTTCATCCGGATCCTGATATACCTCCATC – BHQ1-3’	[24]
EHDV-6 VP2	5′-GATTGTAATAGGAGAGATTAAG-3′	5′-GACCCAAAGCCGCCTGGATT-3′	5′-6-HEX-CGTCAAAATGTCATAACTCGGCAGATGATACC-BHQ1-3′	[24]
